# Study on the Prediction Method of Long-term Benign and Malignant Pulmonary Lesions Based on LSTM

**DOI:** 10.3389/fbioe.2022.791424

**Published:** 2022-03-02

**Authors:** Xindong Liu, Mengnan Wang, Rukhma Aftab

**Affiliations:** ^1^ Faculty of Science, Hong Kong Baptist University, Hong Kong, China; ^2^ College of Information and Computer, Taiyuan University of Technology, Taiyuan, China

**Keywords:** 3D CNNs, time-modulated LSTM, multiscale three-dimensional feature, prediction, characteristics of the fusion, pulmonary lesions

## Abstract

In order to more accurately and comprehensively characterize the changes and development rules of lesion characteristics in pulmonary medical images in different periods, the study was conducted to predict the evolution of pulmonary nodules in the longitudinal dimension of time, and a benign and malignant prediction model of pulmonary lesions in different periods was constructed under multiscale three-dimensional (3D) feature fusion. According to the sequence of computed tomography (CT) images of patients at different stages, 3D interpolation was conducted to generate 3D lung CT images. The 3D features of different size lesions in the lungs were extracted using 3D convolutional neural networks for fusion features. A time-modulated long short-term memory was constructed to predict the benign and malignant lesions by using the improved time-length memory method to learn the feature vectors of lung lesions with temporal and spatial characteristics in different periods. The experiment shows that the area under the curve of the proposed method is 92.71%, which is higher than that of the traditional method.

## 1 Introduction

Because of factors such as smoking, air pollution, and occupational environment, lung cancer has become one of the most malignant tumors that threaten human health and life and has become the number one killer of all cancers (Taillant et al., 2004; Zhang et al., 2018). Global cancer data show that the number of new cases and deaths of lung cancer in the world in 2018 were 2.1 million and 1.8 million, respectively, with the highest morbidity and mortality rates among all cancers. The 5-year survival rate of patients with advanced lung cancer is approximately 16%, but for effective treatment in patients with early-stage disease, the 5-year survival rate can increase by approximately four to five times (Nagaratnam et al., 2018 Cheuk). Pulmonary nodules are an early manifestation of lung cancer, and their benign and malignant predictions are very important for radiologists to carry out cancer staging assessment and individualized clinical treatment planning. With the development of medical imaging technology, the number of computed tomography (CT) images of the lungs continues to increase, but the number of experienced physicians is limited, resulting in the explosive growth of image data and the serious shortage of manual diagnosis. Therefore, computer-aided diagnosis technology is urgently needed (Zhang et al., 2020a) to assist physicians in feature extraction and benign and malignant prediction of lung nodules.

In clinical diagnosis, the objects of lung medical image processing are often limited to the data of the patient in the same period, and the feature vectors of a slice in a certain period are considered in isolation, and the global features with spatial information on the time axis are ignored. In addition, Existing prediction methods, such as medical decision-making systems (Christo et al., 2020) combined with intelligent optimization (Deng et al., 2020; Zhang et al., 2021), are divided into multiobjective (Cui et al., 2020; Cai et al., 2021a) and single-objective optimization (Boudjemaa et al., 2020; Yang et al., 2020). Although the factors considered can be more comprehensive, they mostly rely on artificial features. Because of the limited expressive power of manual features, the prediction effects of existing methods are often unsatisfactory. At the same time, because of the complexity of the growth and evolution of lung nodules in the lung cancer lesion area (Duffy and Field, 2020), the same lesion often has different imaging manifestations at different periods. Among them, the medical imaging data of lesions at different periods contain a large amount of their evolution (development, death)–related information. Lung CT images have blurred edges, low gray values, and difficult-to-express texture information. It is difficult to accurately and comprehensively characterize lung lesions. In recent years, longitudinal prediction methods have been proposed (Santeramo et al., 2018; Oh et al., 2019), and the current research methods are rarely useful in the field of pulmonary medicine, and the existing intelligent diagnosis mostly uses isolated image fragments, which cannot present the entire cycle of the lesion, resulting in the inability to link the characteristics of lung cancer at different periods.

**FIGURE 1:**
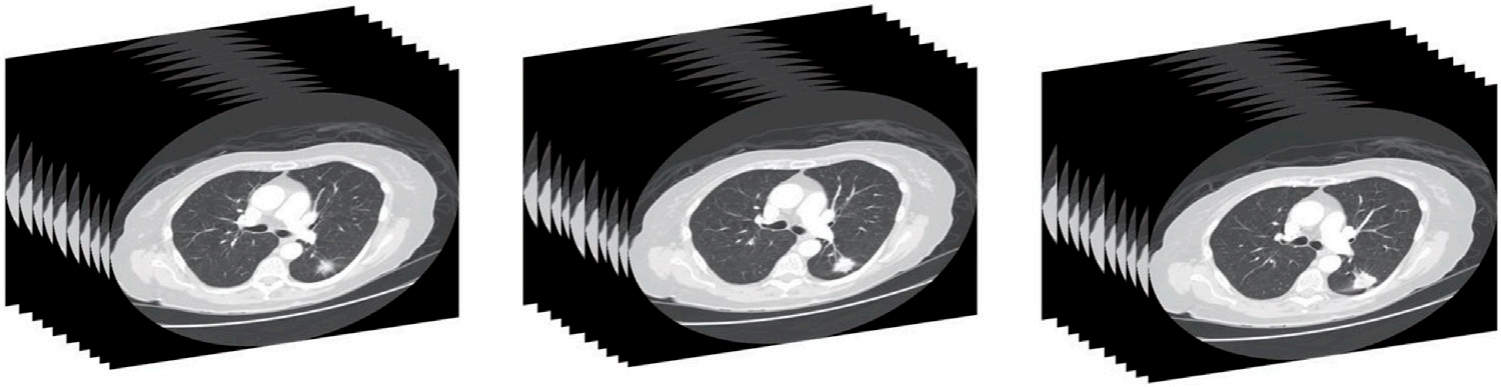
Long-term sequence of lung lesions.

Figure 1 shows the evolution trend of the sequence of long-course lung lesions examined every 3 months in the same patient.

We propose a scheme that uses the latest deep learning techniques (Cui et al., 2021) to extract the depth features of long-term lung CT lesion sequence images for early benign and malignant lung lesion prediction. According to the sequence images of the lesions in each period, make full use of the temporal and spatial information of the image to extract the depth features of the lesions in different periods. According to the characteristics of lung medical images in different periods, the long- and short-term memory model recurrent neural network (RNN) architecture is good for lung lesions. Longitudinal prediction of malignancy provides reliable help for physicians.

The major contributions of this article are as follows:

1) On the lung lesion image data set, RPN was used to extract the candidate region (Ren et al., 2017), and linear interpolation technology was used to obtain the three-dimensional (3D) structure of the candidate region.
2) We propose a novel method to exploit 3D convolutional neural network (CNN) deep network to extract the deep hidden features of long-duration lung lesions; compared with their 2D counterparts, the 3D CNNs can encode richer spatial information and extract more discriminative representations *via* the hierarchical architecture trained with 3D samples.
3) We propose a novel long short-term memory (LSTM) network with time modulation information to propagate the spatial–temporal information between pulmonary lesions adjacent slices for a long period and capture the corresponding long-term dependencies and solve the problem that the input must be the image of lung lesions with equal intervals, thereby predicting the next stage of pulmonary lesion.

## 2 Related Work

### 2.1 Methods of Extracting Medical Image Feature Information

The large amount of information contained in the lesions in each period of medical imaging has important guiding significance for obtaining accurate prediction results, and accurate prediction results also play an important guiding role for doctors’ diagnosis (Hu et al., 2016). For extracting a large amount of information from the lesions, Zhao and Du (2016) used dimensionality reduction technology and deep learning technology, respectively, to extract spectral features and spatial features and used CNN to find space-related features. Bodla et al. (2017) proposed a face recognition method based on deep heterogeneous feature fusion, which uses different deep CNNs (DCNNs) to concatenate the generated features and merge the feature information. Khusnuliawati et al. (2017) proposed that the scale invariant feature transform and local extensive binary pattern should be used for multifeature extraction, and the extracted features should be concatenated and fused in the form of histogram. Xiao et al. (2015) proposed a feature fusion method based on SoftMax regression to perform effective feature fusions by estimating the similarity measure from object to class and the probabilities that each object belongs to a different kind. Shi et al. (2017) put forward a new nonlinear measurement learning method, which uses deep sparse autoencoder feature fusion strategy based on deep network.

### 2.2 Application of Traditional Methods to Time-Series Data

In recent years, scholars have also studied time-series data in medicine. Onisko et al. (2016) analyzed medical time series through Kaplan–Meier estimator Cox proportional hazard regression model and dynamic Bayesian network modeling. Li and Feng (2015) predicted the number of future medical appointments by analyzing the appointment capacity of emergency patients every day and every hour. Cheng et al. (2020) applied a Bayesian nonparametric model based on Gaussian process regression to hospital patient monitoring using clinical covariables and all information provided by laboratory tests and successfully conducted medical intervention. As deep learning has a good advantage in time-series learning, many scholars have applied it to many fields. Chandra (2015) has proposed utilizing RNN to predict collaborative evolution by analyzing time series. Fragkiadaki et al., 2016 proposed an improved RNN model that captures moving body gestures in video for recognition and prediction. Koutník et al. (2014) have introduced a modified clockwork RNN architecture, which divides its hidden layers into separate modules, achieving the processing input of each module at its own time granularity, improving the performance of task tests, and speeding up the network speed.

### 2.3 Application of CNN and Long-Term Memory and LSTM to Time-Series Data

In recent years, CNNs have been successfully used to detect radiological anomalies in medical images, such as ordinary X-rays. LSTMs is a special type of RNN that can classify, process, and predict time series (Graves, 2012; Zhang, 2020). The internal state of the LSTM (also known as cell state or memory) enables the architecture to remember the standard LSTM. The standard LSTM contains memory blocks, which contain memory units. A typical memory block consists of three main components: an input gate controlling the input activation flow of memory cells, an output gate controlling the output activation flow, and a forgetting gate regulating the internal state of cells. The forgetting gate adjusts the amount of information used in the internal state of the previous time step. Santeramo et al. (2018) attempted to automate the analysis of longitudinal medical image data by using the LSTM network to analyses the temporal context of a series of chest radiographs. In the field of breast pathological images, Kooi and Karssemeijer (2017) proposed a region of interest (ROI)–based method to compare plaques aligned at different time points. Although the latter method is slightly improved compared with a single detection method, it depends on specific lesion detection and requires local data.

These algorithms are very effective, but are rarely applied to long-term lung CT image prediction. So far, most studies have used CNNs in individual tests, but abandoned previously available clinical information. One limitation of traditional LSTM is that they implicitly assume equal interval observations, while medical examination is event based, so the sampling is irregular.

LSTMs and more general RNNs do not perform well in time series with irregularly sampled or missing data (Che et al., 2018; Zhang, 2021). Previous attempts to apply LSTMs to irregularly sampled data points focused on accelerating algorithm convergence or reducing short-term memory in an environment with high-resolution sampled data (Baytas et al., 2017). This project set out to explore the performance of LSTM network, which became one of the selection methods of sequence modeling, especially when combined with CNNs for medical image feature extraction (Donahue et al., 2015; Grano and Zhang, 2019). The main advantages of combining CNNs with LSTMs are flexibility and scalability; it allows multiple prior sequences of variable length to be classified using the same network. Longitudinal analysis of images can potentially improve the ability of machine learning algorithms to interpret imaging studies accurately and reliably, thus providing value for medical image processing (Gao et al., 2018).

## 3 Methods

**FIGURE 2:**
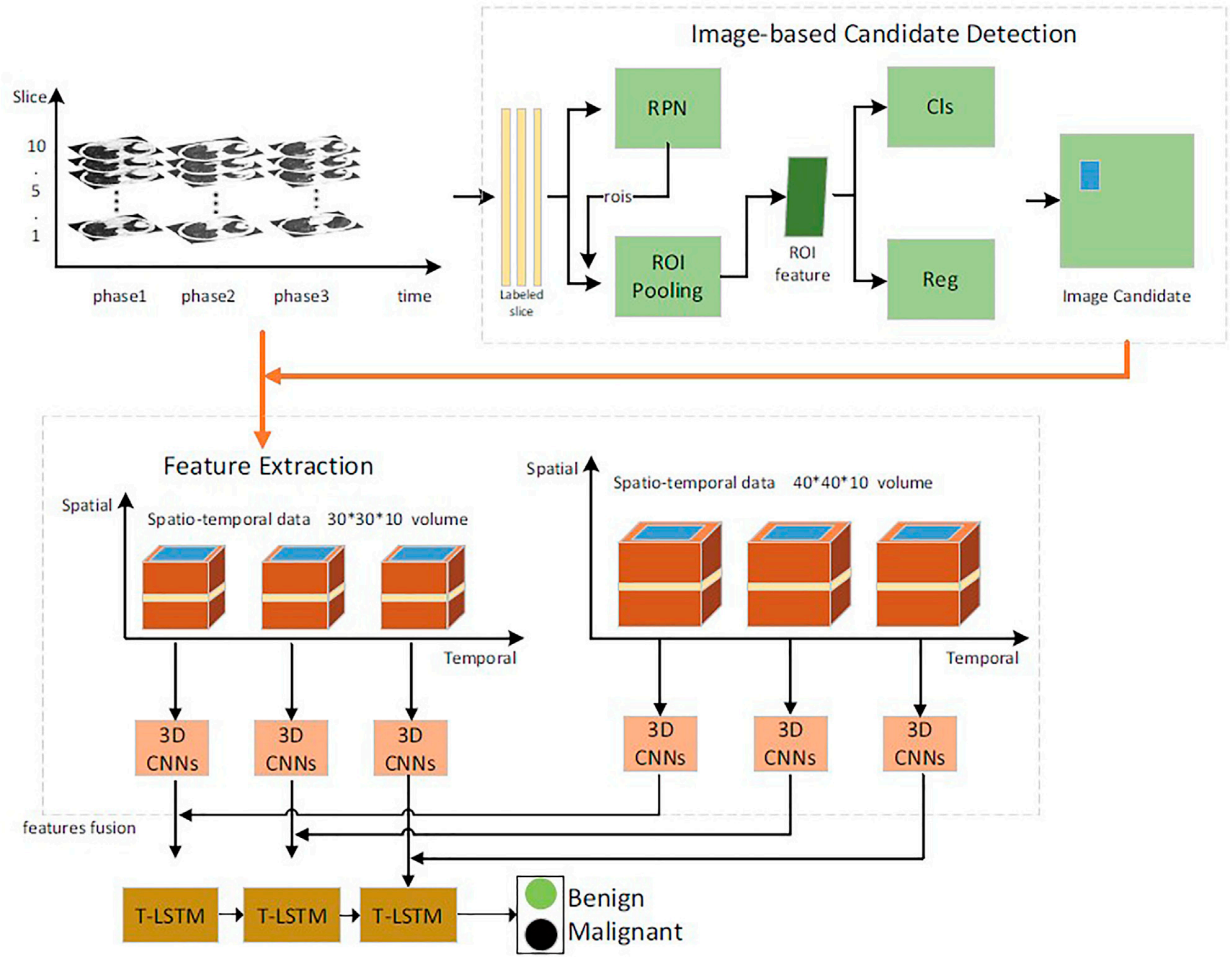
The framework of our proposed network.

In this article, the benign and malignant lung lesions can be predicted by spatiotemporal feature fusion. For CT sequence images of the same patient from the early stage to the diagnosis, a faster Region-CNN (R-CNN) (Shinde et al., 2019) detector was used to generate ROI (Qiang et al., 2015), to extract temporal and spatial features of multilayer context information around pulmonary nodules, and a 3D CNN (Cai et al., 2021b) was used for fusion. Then, the temporal and spatial feature fusion vectors of pulmonary nodules in each period were selected to study the variation trend and relationship of feature vectors in each period by using time-modulated long–short memory network. Finally, the time-modulated LSTM (T-LSTM) model was used to predict the evolution trend of lung lesions over a long period and to determine their malignancy. The overall process is shown in Figure 2.

2D CNN selects ALEXNET network as baseline. CNN architectures for medical imaging usually contain fewer convolutional layers because of the small data sets and input size. The CNN architecture consisted of three convolutional layers and two fully connected layers, where each convolutional layer was followed by a max-pooling layer. In 2D CNN, the kernel moves in two directions. The input and output data of 2D CNN are 3D. It can be mainly used for single image data. In 3D CNN, the kernel moves in three directions. The input and output data of 3D CNN are 4D. It can be mainly used for 3D image data (magnetic resonance imaging, CT scan).

### 3.1 Lung CT Sequence Image Preprocessing

In the diagnosis process of doctors, the focus of observation and research is pulmonary nodules, which are transparent light and shadow with the maximum diameter of no more than 30 mm in the pulmonary parenchyma and occupy only a small part of the CT area of the chest cavity. In order to reduce the interference of other organs and tissues on the diagnosis process of doctors and effectively reduce the algorithm complexity, the lung CT images obtained from The National Lung Screening Trial (NLST) and cooperative hospitals were preprocessed. As the location of pulmonary nodules was not marked in detail in the data set, we adopted a pace–R-CNN detector to detect the target nodules and intercept the ROI-centered peripheral rectangular area to construct the pulmonary nodule data set.

We screened lung CT images of patients followed up for 3 years or more in the NLST data set to construct a long-term data set. The NLST data set marked the section number and approximate location of the most prominent pulmonary nodules in each phase sequence. The pulmonary CT image corresponding to the section number was examined for nodules. ResNet 101 (He et al., 2016) was selected as the backbone network of faster R-CNN. Boundary boxes were defined with five aspect ratios of 1:3, 1:2, 1:1, 2:1, and 3:1 and four scales of 8 × 8, 16 × 16, 32 × 32, and 64 × 64 to cover blocks of different shapes. It is worth noting that the 1:3 and 3:1 aspect ratio settings are due to the presence of pulmonary vascularized lesions, which are critical for the diagnosis of lung cancer.

According to the detection of pulmonary nodules, use a rectangular area with a scale of 30 * 30 or 40 * 40, take the coordinate information of the upper left corner of the detailed annotation rectangle in the lower right corner, cut the first five and the last five rectangular boxes according to the pulmonary nodules with the most obvious coordinate information as the center, and construct a 3D block. When each data set has the same sequence, do the same processing on the CT image, and establish a long-term pulmonary nodule sequence image data set.

### 3.2 Spatiotemporal Feature Extraction

The feature extraction methods of pulmonary lesions can be generally divided into traditional feature extraction methods and deep learning feature extraction methods. Generally speaking, the traditional method of feature extraction can only de-scribe a specific type of information. Deep learning, such as 2D CNN, has achieved good results in image feature extraction and can express high-level semantic information of lesions. However, this solution based on 2D CNN still cannot make full use of the 3D spatial context information of pulmonary nodules to extract the benign and malignant information of pulmonary nodules with temporal and spatial characteristics. Therefore, this article proposes a new method to extract the benign and malignant features of pulmonary nodules from CT sequences using 3D CNNs. Compared with 2D CNN, 3D CNN can encode more spatial information and extract more spatial discrimination information through the hierarchical structure of 3D sample training.

Features extracted by DCNN can represent the inherent semantic information of images (Kamnitsas et al., 2016). With the emergence of deep neural networks in computer vision, 3D CNN has developed rapidly in the past few years. Although 3D medical data are very common and popular in clinical practice, 3D CNN is still in its infancy in medical application. Furthermore, the hyperparameter adjustment of thousands of filters on large data sets is still an important challenge. To alleviate this problem, migrating pretrained 3D CNN to specific application scenarios is a very efficient and simple solution (Aaron et al., 2018).

**FIGURE 3:**
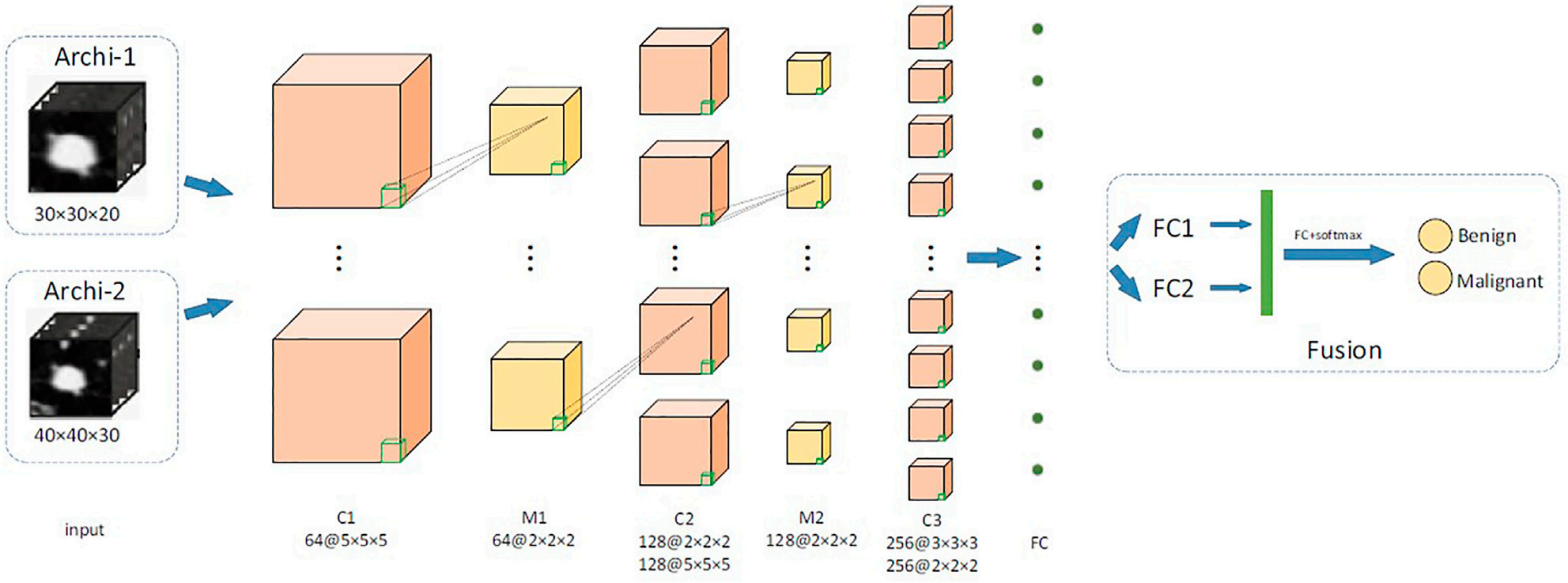
The main network structure of multiscale 3D CNN framework. C is the 3D convolutional layer; MP represents the 3D maximum pooling layer, whereas FC is the full connection layer.

We proposed a two-channel network, which is suitable for input of different sizes. The main structure of our multilevel 3D CNN framework is shown in Figure 3. Each network has four convolutional layers. Both cnn-30 and cnn-40 contain a fully connected layer. After each hidden layer, a batch normalization layer is inserted to ensure a higher learning rate and reduce overfitting, and a dropout layer is added to further reduce the overfitting performance.

The two architectures, respectively, output the 2D classification prediction of nodule or nonnodule by SoftMax in the upper layer and a 256-D feature vector from the last hidden layer. Their outputs are then combined into a single classification result of a given original 3D volume. This feature is used for feature fusion and for predicting classification of pulmonary nodules. We used data fusion techniques to, namely, late fusion. The two features from the last hidden layer of CNN are connected into a complete feature vector and sent to the prediction module. Table 1 details the network configuration.

**TABLE 1:** Architecture of the multilevel contextual 3D CNNs.

Archi-1			Archi-2		
Layer	Kernel	Channel	Layer	Kernel	Channel
Input	—	1	Input	—	1
C1	5 × 5 × 5	64	C1	5 × 5 × 5	64
M1	2 × 2 × 2	64	M1	2 × 2 × 2	64
C2	2 × 2 × 2	128	C2	5 × 5 × 5	128
M2	2 × 2 × 2	128	M2	2 × 2 × 2	128
C3	3 × 3 × 3	256	C3	2 × 2 × 2	256
FC1		256	FC1	—	256

A batch of 3D training samples are expressed as $\left(x^{1},y^{1}\right)\ldots \left(x^{\text{i}},y^{\text{i}}\right)\ldots \ldots \left(x^{m},y^{m}\right)$ , where *m* is the number of samples, $x^{i}$ is the input sample, and $y^{i}$ is the real label corresponding to the sample. $y^{i}\in \left[0,1\right]$ , where 0 represents benign nodule and one represents malignant nodule. ${\text{p}}^{i}$ is the probability of prediction, and θ represents all trainable parameters in the model. In this article, the weight factor of the right of use, α∈[0,1] , and the adjustable focus parameter, γ≥0 , are used to solve the class imbalance problem, and the attention is focused on the sample of more complex training situations. The population objective function is the average value of the sample loss, as shown in Eq. 1, minimizing J(θ) by optimizing network parameters.

$$J\left(\theta \right)=-\frac{1}{m}\left[\alpha y^{i}{\left(1-p\left(\theta \right)\right)}^{\gamma }\log\left(p^{i}\left(\theta \right)\right)+\left(1-\alpha \right)\left(1-y^{i}\right)\left(p^{i}{\left(\theta \right)}^{\gamma }\log\left(1-p^{i}\left(\theta \right)\right)\right)\right]$$ (1)

### 3.3 Long-Term Lung Lesion Prediction Based on the T-LSTM Model

In this article, the long-term pulmonary nodules sequence image data set prepared in Section 3.1 was used to construct a long-term pulmonary nodule benign and malignant prediction model. LSTM and RNN are deep network architecture. The connection between hidden units forms a directed cycle. The feedback loop enables the network to save the previous hidden state information as internal memory. Therefore, RNNs are preferred for problems where the system needs to store and update the context information (Li et al., 2020). Hidden Markov model (HMM) and other methods are also used for similar purposes. However, RNN has its unique characteristics, which is different from traditional methods (such as HMM). For example, RNN can deal with variable length sequences without the assumption of Markov property. In addition, in principle, the information entered in the past can be saved in memory without being limited by the past time. However, in practice, the optimization of long-term dependence is not always possible. Because when the gradient value becomes too small and too large, the gradient value will disappear and explode. In order to merge long-term dependencies without violating the optimization process, a variant of RNN has been proposed. One popular variant is LSTM, which can handle long-term dependencies using gated structures (Huimei et al., 2020).

However, traditional LSTMs are not suitable for our task because the time between consecutive follow-up of patients is variable (Zhang et al., 2020b), and they have no mechanism to explicitly model the arrival time of each observation (Baytas et al., 2017). In fact, LSTM and, more generally, RNN have been shown to perform poorly in time series with irregular sampling data or lack of values (Che et al., 2018). A previous study attempted to use LSTM for irregular sampling data points mainly focused on accelerating the convergence speed of the algorithm or reducing short-term memory in the setting with high-resolution sampling data.

For the first time, we propose a temporal information enhancing LSTM neural networks (T-LSTM) that combine recurrent time labels with RNNs, which makes the best use of the temporal features to improve the accuracy of short-term prediction. And the Long-term lung lesion prediction algorithm in T-LSTM is shown as Algorithm 1.

**FIGURE 4:**
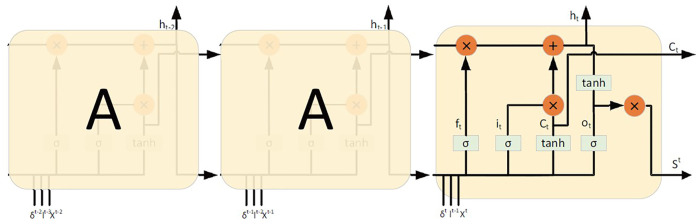
T-LSTM cell.

To solve these problems, we introduce two simple modifications to the standard LSTM architecture, called T-LSTM, both of which explicitly use the input-related time index. In the architecture proposed in this article, all images of a given patient are first processed by CNN architecture, which extracts a set of image features, denoted by *X* ^ˆ*t*^, at each time step. The LSTM takes as inputs $l_{i}^{t-1}$ , that is, the radiological labels describing the images acquired at the previous time step, the current image features ${\hat{X}}_{i}^{t}$ , and the time lapse between $X_{i}^{t-1}$ and $X_{i}^{t}$ , which we denote as:

For the last image in the sequence, the LSTM predicts the image labels $l_{i}^{t}$ , called ${\text{y}}_{i}^{t}$ .The cell structure of T-LSTM is shown in Figure 4 The equations below define the T-LSTM unit:

$$f_{t}=\sigma \left(W_{fl}*l^{t-1}+W_{fx}*{\hat{X}}^{t}+W_{fj}*\delta^{t}+b_{f}\right)$$ (2)

$$i_{t}=\sigma \left(W_{il}*l^{t-1}+W_{ix}*{\hat{X}}^{t}+W_{ij}*\delta^{t}+b_{i}\right)$$ (3)

$$o_{t}=\sigma \left(W_{ol}*l^{t-1}+W_{ox}*{\hat{X}}^{t}+W_{oj}*\delta^{t}+b_{o}\right)$$ (4)

$$c_{t}=\tanh\left(W_{cl}*l^{t-1}+W_{cx}*{\hat{X}}^{t}+W_{cj}*\delta^{t}+b_{c}\right)$$ (5)

$$h_{t}=f_{t}*h_{t-1}+i_{t}*c_{i}$$ (6)

$$y^{t}=o_{t}*\tanh\left(h_{t}\right)$$ (7)

Algorithm 1 Long-term lung lesion prediction algorithm for T-LSTM.

Input: fusions of pulmonary nodules at different periods of the same patient *X* ^ˆ*t*^, *t* = 1, 2, 3;
Output: The results of classification {*0,1*}.
Step 1:
When calculating C0, the first implied state, Ct−1 is needed, but it does not exist, so it is set to 0.
Step 2:
Calculate the input gate, such as Eq. 3, including the benign and malignant label of the lesion sequence image at time *t*, the input of the feature vector of the lesion sequence image at time *t*, and the time interval between *t*−1 and *t*. The activation function is calculated after summation.
Step 3:
The forgetting gate was calculated as Eq. 2, including the benign and malignant labels of the lesion sequence image at time *t*, the input of the feature vector of the lesion sequence image at time *t*, and the time interval between *t*−1 and *t*. The activation function is calculated after summation.
Step 4:
The output gate is calculated as Eq. 4, which includes the benign and malignant labels of the lesion sequence image at time *t*, the input of the feature vector of the lesion sequence image at time *t*, and the cumulative sum of the time intervals of *t*−1 and *t*, and then the activation function is calculated.
Step 5:
The computational memory unit (the first layer is not calculated), as shown in Eq. 5, contains the benign and malignant labels of the lesion sequence image at time *t*, the input of the feature vector of the lesion sequence image at time *t*, and the time interval between *t*−1 and *t*. The activation function is calculated after summation.
Step 6:
Calculation of implicit elements, such as Eq. 6.
Step 7:
Repeat steps Eqs 2–6 to calculate the input and output of each layer by layer.

## 4 Experiments and Results

### 4.1 Data Sets

In order to train and classify CNN, we used two labeled lung data sets. One is the NLST data set, and the other is the provided cooperative hospital data set.

*NLST* (The Landmark National Lung Screening Trial) data set. The NLST is a randomized, multisite trial that examined lung cancer–specific mortality among participants in an asymptomatic high-risk cohort. Subjects underwent screening with the use of low-dose CT or chest X-ray. More than 53,000 participants each underwent three annual screenings from 2002 to 2007 (approximately 25,500 in the LDCT study arm), with follow-up postscreening through 2009. Lung cancers identified as pulmonary nodules were confirmed by diagnostic procedures (e.g., biopsy, cytology); participants with confirmed lung cancer were subsequently removed from the trial for treatment through 2009. NLST contains 421 CT scans annotated by four radiological experts voxel-wise.

The cooperative hospital had CT images of the lungs of 267 patients, a total of 1,837 cases. The pulmonary CT images of the cooperation hospital were taken from the positron emission tomography (PET)/CT center of a hospital in Shanxi Province in January 2011 and January 2017. The medical equipment used was Discovery ST16 PET/CT of GE. The CT image acquisition parameters were as follows: 150 mA, 140 kV, layer thickness 3.75 mm, and image resolution 512 × 512. Under the diagnosis of two professional radiologists, the nodule location was marked, and all cases were marked with 1 and 0, respectively.

### 4.2 Input Description

We determined the size of the receptive field used in our framework by analyzing the size distribution of pulmonary nodules. Firstly, we observed that the diameter density peak of small nodules was about 9 voxels in X and Y dimensions and about 4 voxels in Z dimension. We set the first network, Archi-1, with an acceptance domain of 30 × 30 × 10 (voxels). This receiving domain can contain small pulmonary nodules in the appropriate context, and it covers 85% of all nodules in the data set. This can be performed well under normal circumstances, most often in patients. The purpose of this window size is to provide rich background information for small nodules and appropriate background information for medium-sized lesions. For some large nodules, it can usually include their main parts and exclude some marginal areas. Finally, we constructed an overall acceptance domain of 40 × 40 × 10. According to our statistical analysis, the boundary of this model is more than 99% of nodules, except for several outliers.

### 4.3 Classification Accuracy Comparison of 3D CNN Feature Extraction Methods With Different Parameters

This article adopts the method of uniform random sampling; the NLST data set is divided into training set validation set and test set. Three parts will be 1 over 10 of the NLST data set as a test set; the rest of the data according to speak is divided into training set and test set because the model in clinical practice needs to detect significant differences of data and training data, so we use team hospital to provide the data set and the NLST test set as a test set to select the training program.

Training process, from the positive and negative sample dropout layer and maxnorm regularization, weight initialization, data expansion four aspects to experiment on the two-validation set to explore the four aspects of the influence of different combination for the model to detect lung nodules on the NLST test and cooperation hospital test sets of prediction results, and the network parameters as shown in Tables 2, 3, which define the sensitivity, specificity, accuracy, and *F* score of the four parameters to evaluate the classification effect of nodules. The dropout rates are 1:20, 1:10, 1:5, 1:3, and 1:2.

**TABLE 2:** The classification results and network parameters on NLST test set.

Method	Sensitivity	Specificity	Accuracy	F1 score
1:20 + Dropout	0.801	0.999	0.905	0.891
1:20 + Dropout + Maxnorm	0.752	0.998	0.883	0.863
1:10 + Dropout + Maxnorm	0.861	0.998	0.921	0.904
1:5 + Dropout + Maxnorm	0.908	0.994	0.949	0.923
1:3 + Dropout + Maxnorm	0.917	0.994	0.953	0.913
1:2 + Dropout + Maxnorm	0.924	0.991	0.957	0.924
1:2 + Dropout + Maxnorm + Lecun	0.932	**0.989**	0.954	0.917
1:2 + Dropout + Maxnorm + Lecun + Aug	0.943	**0.985**	**0.965**	0.929

The bold values is the best performance.

**TABLE 3:** The classification results and network parameters on cooperative hospital test set.

Method	Sensitivity	Specificity	Accuracy	F1score
1:20 + Dropout	0.711	0.908	0.815	0.864
1:20 + Dropout + Maxnorm	0.705	0.900	0.800	0.848
1:10 + Dropout + Maxnorm	0.721	0.908	0.817	0.864
1:5 + Dropout + Maxnorm	0.717	0.907	0.813	0.871
1:3 + Dropout + Maxnorm	0.709	0.886	0.799	0.862
1:2 + Dropout + Maxnorm	0.698	0.870	0.779	0.952
1:2 + Dropout + Maxnorm + Lecun	0.760	0.943	0.851	0.878
1:2 + Dropout + Maxnorm + Lecun + Aug	0.814	**0.946**	0.880	0.901

The bold values is the best performance.

First, it can be seen from Tables 2, 3, when the samples are rare, even in the process of testing, all samples to sample more than one, and the same accuracy can be higher. Thus, the balance of positive and negative samples in the training is very important in this article. The main purpose of this model from the hundreds of thousands of pieces of chest CT image sequence forecasts suggestive of benign and malignant lesion area is for the doctor to prescreen in the end. The bold values is the best performance.

It can be seen from Tables 4 and 5 that the accuracy of the basic RNN tanh-RNN can reach 87.1%, which verifies that the RNN has the ability of learning and discriminating features. Support Vector Machines (SVM) is a traditional feature extraction and classification method. As it is unable to learn deep hidden features and their existing relationships, its accuracy is relatively low. However, the T-LSTM network proposed in this article is higher than RNN, which proves that considering the relevant continuous changes of things is helpful to further improve the accuracy of prediction. The bold values is the best performance.

**TABLE 4:** Comparison of prediction performance of different methods.

Algorithm	ACC (%)	Pre (%)	Rec (%)	*F* score (s)
SVM	0.812	0.818	0.813	0.819
tanh-RNN	0.871	0.936	0.778	0.874
LSTM	0.911	0.943	0.875	0.903
T-LSTM	**0.928**	**0.948**	**0.927**	**0.938**

The bold values is the best performance.

**TABLE 5:** Results for all models, AUROC, and specificity at sensitivity (SPC@SEN) of 0.87, with 95% confidence interval (CI) displayed in brackets.

	AUROC [CI]	SPC@SEN 0.87 [CI]
LSTM	0.82 [0.732–0.821]	0.62 [0.401–0.705]
xgb	0.84 [0.789–0.880]	0.75 [0.534–0.721]
BI-LSTM	0.90 [0.802–0.908]	0.72 [0.543–0.813]
T-LSTM	**0.93[0.825–0.921]**	0.78 [0.561–0.921]
RNN	0.88 [0.744–0.851]	0.59 [0.371–0.752]

*p < 1e-6 compared with RNN. The bold values is the best performance.

### 4.4 Discussion of the Number of LSTM Layers

The number of network layers directly affects the ability of the network to extract the characteristics of lung nodules. Theoretically, the more hidden layers, the more complex the network structure, making the network have a strong feature extraction ability, and the higher the accuracy. However, blindly increasing the number of network layers will result in increased difficulty of network training, greatly prolonged learning time, and poor accuracy. In this article, the network structure with different hidden layers is studied to ensure that other parameters of the network remain unchanged, and the average value is calculated 10 times per iteration. Generally speaking, the more layers of LSTM module, the stronger the learning ability of higher-level time representation. At the same time, a layer of ordinary neural network is added to reduce the dimension of the output results.

**FIGURE 5:**
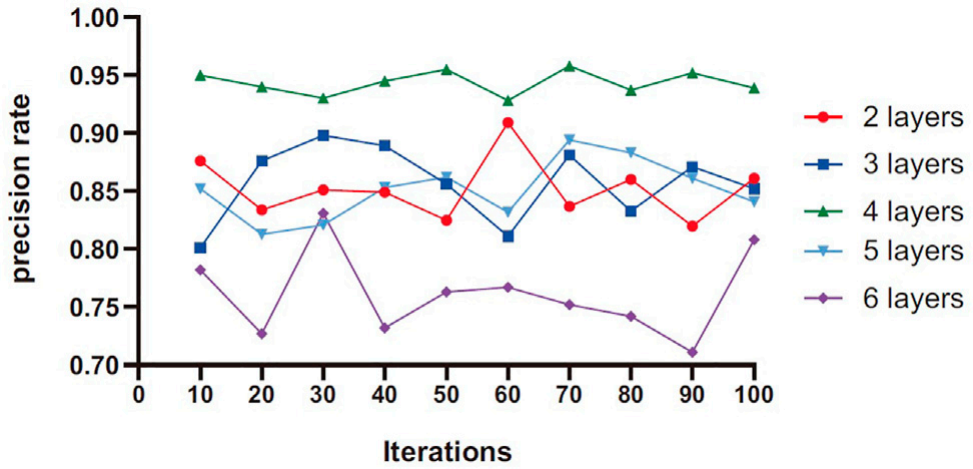
Layer number experimental result diagram.

As can be seen from Figure 5, the prediction accuracy increases first and then decreases with the increase of the number of network layers. When the number of network layers is 4, the overall accuracy is higher than other values. When the number of layers in the network is 6, because the number of layers is too deep and difficult to converge, and at the same time, the high-level abstract feature information weakens the differentiation of benign and malignant nodules, the result will fall into the local extreme value, and the accuracy is reduced.

### 4.5 Comparison of Convergence Effect of T-LSTM

**FIGURE 6:**
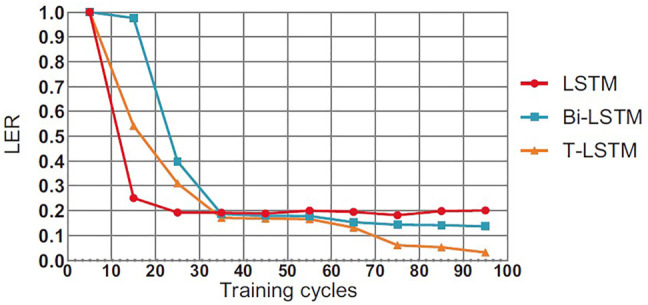
Comparison of convergence LER results between T-LSTM and BI-LSTM and LSTM.

**FIGURE 7:**
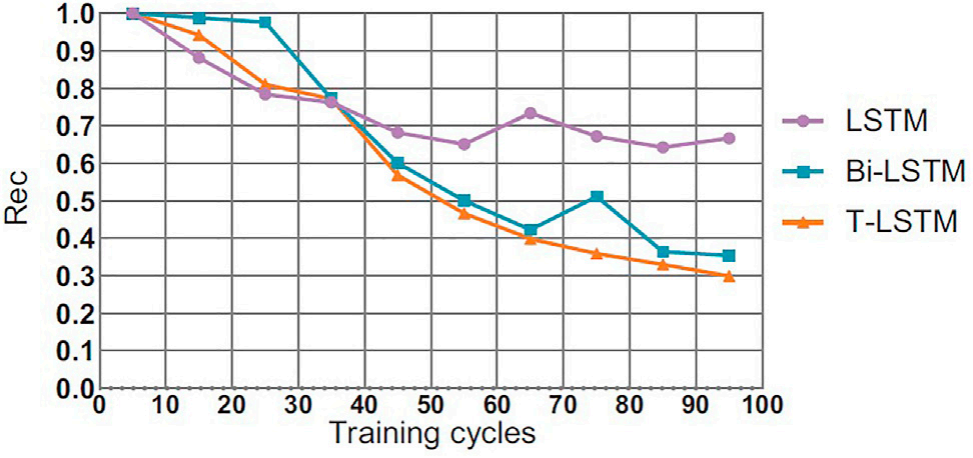
Comparison of convergence Rec results between T-LSTM and BI-LSTM and LSTM.

**FIGURE 8:**
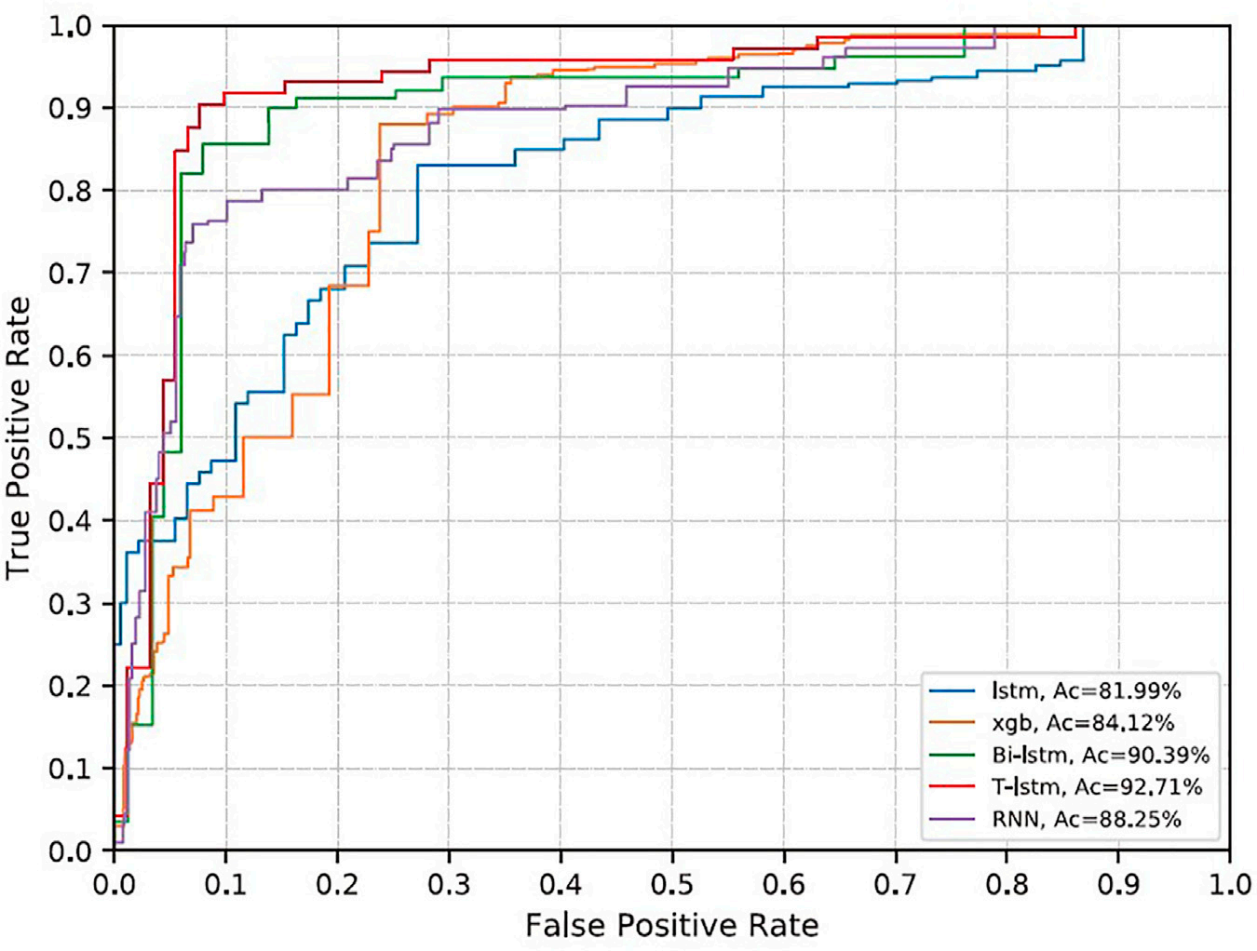
ROC curve of each model. Blue is LSTM; orange is gradient boost (xgb); green is BiLSTM; red is T-LSTM; and purple is RNN.

This section will compare the performance of the T-LSTM and the Bi-directional Long Short-Term Memory (BI-LSTM) LSTM in the training process. In theory, the BI-LSTM model takes about twice as much time as the LSTM because of its bidirectional structure. The single-cycle time of the T-LSTM is approximately 1.4 times as much as the LSTM due to the fact that the input data of the T-LSTM are more than those of the LSTM as shown in Figures 6–8, in the training process of neural network, although the LSTM converged faster than T-LSTM and BI-LSTM at the beginning; the time of BI-LSTM was only 1.5 times that of LSTM and that of T-LSTM was only 1.2 times that of LSTM due to the impact of data reading speed and other factors. After some periodic training, when LSTM and BI-LSTM gradually approach a constant value, T-LSTM can continue to converge. From the perspective of recognition effect, T-LSTM performs better than the other two. From the perspective of model convergence and recognition effect, the validity of time-modulated recursive neural network structure is proven.

### 4.6 Comparison of Prediction Rates Among Different Classifiers

The RNN classifier does not add *a priori* knowledge. The AUC under the receiver operating characteristic (ROC curve; AUROC) obtained on the evaluation set is 0.88, the sensitivity is 0.87, and the specificity is 0.59. LSTM did not improve the accuracy and decreased slightly compared with RNN (Table 4). BI-LSTM increased AUROC to 0.90 and specificity to 0.64, which was not statistically significant. The improvement obtained by gradient boosting was more significant (AUROC 0.84, specificity 0.75, *p* < 1e−6). The T-LSTM network further improved the performance, with AUROC of 0.93, specificity of 0.78, and sensitivity of 0.87. The ROC curves of the five classifiers are given in Figure 8.

We can see that because the ability of network to learn from image sequences is limited by depth, RNN is not as good as BI-LSTM. In future work, we intend to validate our results on a larger evaluation set. The further improvement of this work is to train 3D CNN and T-LSTM networks at the same time to realize the joint optimization of the whole classification architecture. In addition, we will consider the role of clinical information in guiding classification. Finally, we can also evaluate the effect of using multiple *a priori* knowledge or neighborhood knowledge in the training set. In conclusion, combining long-time sequence image research in the deep learning analysis framework can improve the classification performance and enhance radiologists’ confidence in the reliability of decision support technology.

## 5 Discussion

It is reported that deep learning algorithm can achieve high performance in medical image classification task (Kooi and Karssemeijer, 2017; Ribli et al., 2018). However, the current algorithm is still lower than the average level of human radiologists in real-world data. One explanation for this gap is that radiologists add additional information to their diagnostic analysis, such as nonimage clinical information and patient specific information. We address the latter by allowing our algorithm to analyze current and previous studies. Most of the literatures in this field do not take into account the relevant characteristics and information of patient time series, so it is difficult to accurately compare the performance. On different data sets, AUROC values for cancer classification ranged from 0.79 to 0.95. The AUROCs with time information and without time information are 0.82 and 0.93, respectively, which is different from the related work (Kooi and Karssemeijer, 2017), reflecting the significant benefits of using previous studies. The advantage of our method is that it only needs to comprehensively label each pulmonary nodule without expensive local lesion description. The experimental results show that it is not enough to simply classify the images separately; only by training the classification algorithm on the long-time sequence image can it be improved.

It can be seen in Table 4, there are two serious problems in RNN: gradient explosion and gradient disappearance; thus, the follow-up training results are not very good. LSTM improves the gradient updating process, which is mainly generated by the accumulation of the output of each gate, so as to avoid the problems of gradient explosion and gradient disappearance caused by accumulation and multiplication such as RNN. Bidirectional LSTM is actually the integration of two LSTM (forward and backward) to enable them to extract information from the above and below at the same time. The main integration methods are direct splicing concatenated and weighted summation. Adding nonlinear characteristics can also fit the data better. The training system that provides the highest performance is T-LSTM, which trains based on features of 3D CNN extracted. T-LSTM solution is also scalable for analyzing multiple *a priori* sequences, and we will further study how to increase scalability and robustness in the future. In Table 2, there is a lower probability of specificity after data enhancement than without data enhancement, which may be related to the setting of data enhancement parameters. It can reduce the overfitting of data, but also depends on enhanced effects and methods. Although the proposed method has a certain reduction, it is within a reasonable range. In Figures 5, 6, the convergence speed of the proposed method is slower than that of LSTM at the beginning, but it can achieve the best convergence effect after 35 iterations. This shows that our method can realize effective processing and analysis for data with more time information.

However, there are still some limitations. If the time span of LSTM is very large and the network is very deep, this calculation will be very time-consuming. Meanwhile, this network structure also has certain limitations in efficiency and scalability. In addition, there is the issue of data size. An LSTM is a neural network and like any neural network requires a large amount of data to be trained on properly. The information with a time series needs to traverse all the cells before entering the current processing unit. This generates vanishing gradient. LSTM does not completely solve this problem. The methods proposed in this article tend to do better on unstable time series with more fixed components because of their inherent ability to quickly adapt to sharp changes in trends. However, this method can only make short-term prediction, and remote prediction may be invalid. This is also one of the limitations of the proposed method. In future work, we will consider how to better learn on medical small sample data sets. And we will try to improve the robustness and generalization of the algorithm so that the model can be used in more different scenarios and environments.

## 6 Conclusion

In this article, we have used and substantially extended LSTM in the 3D spatial–temporal domain for the task of modeling 3D longitudinal pulmonary nodule data. The novel 3D CNNs and T-LSTM network jointly learn the interslice structures, the interslice 3D contexts, and the temporal dynamics. Quantitative results of notably higher accuracies than the original RNN are reported, using several metrics on predicting the future tumor volumes. Compared with the most recent 2D + time deep learning–based tumor growth prediction models (Missrie et al., 2018; Audrey et al., 2019), our new approach directly works on 3D imaging space and incorporates clinical factors in an end-to-end trainable manner. This method can also detect the benign and malignant pulmonary nodules. Our experiments are conducted on the largest longitudinal lung data set (421 patients) to date and demonstrate the validity of our proposed method. This method enables efficient and effective 3D medical image segmentation with only sparse manual image annotations required. The presented prediction model can potentially enable other applications of medical sequence imaging applications. Gradient extinction can be remedied with the LSTM module, which is currently considered a multiswitched gateway, a bit like ResNet. Because LSTM can bypass some cells and memorize long steps, LSTM can solve the gradient disappearance problem to some extent. This method can provide technical support for processing medical image data or bioinformatics data with time information in the future.

## Data Availability

The original contributions presented in the study are included in the article/Supplementary Material, further inquiries can be directed to the corresponding author.
